# Is Phthisis Self-limiting?

**Published:** 1880-01

**Authors:** Wm. Porter


### Selections.

---

IS PHTHISIS SELF-LIMITING? By WM. PORTER, A.M., M.D., Read before the St. Louis Medical Society, Oct. 1877. From advance proof pages furnished by the author.

It is well known that at a recent meeting of the New York Academy of Medicine (May 1879), Prof. Flint asserted his belief in the self-limitation of phthisis (*Arch. of Med.*) This assertion was made so forcibly and sustained by such seemingly positive proof that it has thus far been received without recorded dissent. It being a subject of vital importance, with all respect for the learned author, we will examine his position from his own stand-point, and cite the cases which he has noted. The deductions that follow will thus be made upon ground already chosen.

Let us, then, gentlemen, receive the evidence purely upon its merits, forgetting, if possible, the high authority which we call in question. It is, moreover, right that a decision be reached in this matter, for if phthisis be self-limiting, this element must necessarily affect the result in no small degree; but if it is not, then must we look to therapeutic force and hygienic conditions for success.

At the outset, however, I cannot but echo Andrew Clarke's just tribute to our author, for he is "one from whom I have learned much; one whose acute powers of observation, whose largeness of experience, deserve the warmest gratitude of every student of pulmonary pathology." We revere and honor Flint; therefore, we shall deal freely and directly with his conclusions.

What is meant by self-limitation? So far as our argument is concerned, the definition given by Prof. Flint is well adapted. "A disease is self-limited when it ends in recovery, irrespective of extrinsic influence derived from either hygiene or therapeutics."

We might almost rest our case here, for it will be at once conceded that the rule is that phthisis does not end in recovery, irrespective of extrinsic influence. To this rule there are few, if any, exceptions—certainly too few to affect the question. To these exceptions, however, our author points, and says they "are amply sufficient to substantiate the statement that in certain cases pneumonic phthisis ceases to be progressive, and may end in recovery from self-limitation." I will endeavor to prove to you, gentlemen, that phthisis is not, and cannot be, from its very nature, a self-limiting disease; and will ask you to examine with me briefly, first, whatever in the causation and pathology of phthisis relates to the subject; and, second, the clinical evidence adduced by Prof. Flint to substantiate his position.

Let us at the outset look for a working definition of phthisis. Flint, in the discourse mentioned, considers the "term pneumonic phthisis or pulmonary consumption as applicable to all cases of phthisical disease, exclusive of acute tuberculosis or interstitial pneumonia." Very true. What, then, is phthisical disease? A wasting, a constitutional disease, "a progression of symptoms, characterized," as Andrew Clarke has it (*New York Medical Record*, Dec. 14, 1878) by an ulcerative or suppurative destruction of a more or less circumscribed non-malignant deposit in the lung." The great factor back of all local manifestation is, then, the phthisical cachexia. Flint himself (Prac. Med. p. 289), admits the pathological fact that the pulmonary invasion is "the local expression of a general or constitutional morbid condition, the latter being the essential disease." He further says, "the great object of treatment, therefore, is the removal of this constitutional morbid condition or the tubercular cachexia. Measures addressed to the pulmonary affection are of secondary importance." How, gentlemen, can we believe that pulmonary phthisis, the local manifestation, is self-limiting, when back of it we have the tubercular cachexia? The author having taught that phthisis is the local expression of a cachexia, yet self-limiting, the deduction must be that the cachexia—this tubercular cachexia—is self-limiting also; in other words, that this condition, with its sequences, has an "intrinsic tendency to recovery irrespective of extrinsic influences derived from either hygiene or therapeutics." Is it any wonder that we turn from the *ipse dixit*, and call for proof ere we subscribe to this doctrine?

Let us glance at some of the causes of phthisis, and see if we can relegate to them a self-limiting tendency. Among these we have inheritability, age, climate, habits of life, poor food and exposure. Now, if from any of these causes—and these are the principal ones—a man has phthisis, is it reasonable to expect a limit from intrinsic influence? And if the man recovers, the cause being removed, is it not to this rather than to self-limitation that the result is due? When a patient with an inherited tendency to phthisis is so strengthened as to resist the advance of the disease, or by care and prudence lives beyond the dangerous decade; or weakened by an effeminating, changeable climate, seeks a better one, or exchanges a hurtful occupation for one more favoroble—there is a cause for the stay of the disease more tangible than "self-limitation." To such a cause will we find most, if not all, recoveries from phthisis are due.

A few words regarding the pathological characteristics of phthisis. Here I cannot serve my purpose better than to again quote from Prof. Flint (Prac. Med., p. 280): "The fact to be especially borne in mind is that pulmonary tuberculosis is not primarily and essentially an affection of the lungs. The tuberculous products proceed from a prior morbid condition of the system. It is a rational inference that a vice of assimilation is involved in the existing cachexia." Putting aside all disputed points as to the relation of true tubercle to phthisis (Prof. Flint using the terms phthisis and pulmonary tuberculosis in the same sense—(Prac. Med., p. 271), we have this succession which all admit—cachexia, mal-assimilation, pulmonary invasion. The latter impairs the respiratory function, and thus reacting upon the assimilative, the cachexia is further determined. The patient is thus inclosed in a morbid circle which can only be broken from without. Limitation must result from either repressing the cachexia, restoring assimilation, effecting retrogression of the pulmonary invasion, or all of these together. Tell me what is there intrinsic in this vicious circle which will cause it to break itself? The rather, each link becoming its own cause, grows stronger through the others, and without extrinsic influence the disease progresses. In the very nature of cause and effect it cannot limit itself.

If, then, what we know of the causation and character of phthisis is opposed to the idea of self-limitation, let us examine the clinical evidence adduced in support of this theory.

Prof. Flint, in his well-known work on Phthisis (1875), records 670 cases of this disease, among which were 75 in which either recovery took place or the disease became latent; and his recent paper is founded upon their histories. Now, gentlemen, it is to those 75 cases that we must look for all the proof that has been given of the self-limitation of phthisis. By these, thus far, must the proposition stand or fall. We find that in 31 of these cases the statement is merely that "the disease ceased to progress for at least several months, and in the majority of cases for several years." By reference to the record we find that the last examination of each gave evidence that the disease was still present—latent in some, as may occur in phthisis, but not self-limited; for "a disease is self-limited when it ends in recovery," etc., and these had not recovered. As according to our author's own definition 31 of these cases have no definite bearing upon the point in question, we are restricted to the study of the remaining 44.

As self-limitation is independent "of extrinsic influence, derived from either hygiene or therapeutics," we at once decline the evidence of twenty-one of the forty-four cases, for in all of these pertinent and generally persistent treatment was pursued. Moreover, *three* of these cases subseqently proved fatal, and the last examination showed that at least a third of them had still physical signs of phthisis. We must object to these twenty-one histories as pertinent. The interest now centers in but twenty-three. "In fifteen of these cases hygienic measures constituted the treatment;" but these measures were of such a character as would lead us to hope for favorable results, viz., changes of business, out-door life, rest, sea voyages, change of climate, etc. These are potent aids, for as Flint says (Prac. Med., p. 290), "out of door life is of all measures most important." Now, to prove a disease self-limiting, we must eliminate whatever can be reasonably traced to "either hygiene or therapeutics." These fifteen cases were given the advantage of favorable hygienic condition, and who shall say they would have recovered without these conditions? Having made use of that remedy which of all others has been found efficacious in phthisis, these fifteen cases are certainly not examples of self-limitation.

I have refrained from occupying your time with the details of the author's cases, which, in accordance with his own definition of limitation, have been refused as evidence. The history of these is fully given in his work on Phthisis, and I have endeavored to deal fairly and justly with the record.

Let us now apply the test to the eight cases which alone remain. These are numbered 1, 4, 7, 8, 14, 20, 23, and 24 (Phthisis, p. 187, *et seq.*) and are the only ones of which the author says "there was no medicinal treatment of importance, and no material change in the habits of life, the recovery taking place purely from an intrinsic tendency."

CASE I is that of a farmer who, having in the winter of 1842-'43, expectorated what were thought to be pulmonary calculi, was examined in June, 1843; "the only physical sign noted was feebleness of the respiratory murmur." He was in excellent health thirteen years after. "Prior to the development of the disease the patient had worked very hard on a farm. He left home for several weeks, and after relinquishing severe labor, engaged in buying and selling new lands in Illinois, a business which required much out of door life."

CASE VII is that of a constable, examined in April, 1856. Six years before he had had a hæmorrhage, and shortly afterward recurrent hæmoptysis for ten days. During the following year Prof. Flint met this man from time to time on the street, and he seemed to be in good health. As this man evidently had phthisis for six years prior to examination, what reason is there, in the absence of a later examination, to suppose that he had entirely recovered during the succeeding year? The statement that he seemed well will apply, at times, to many cases of chronic phthisis. "There is no further record of this case. The treatment consisted of cod liver oil for six weeks, generous living, the use of malt liquors, and out of door life. Very good extrinsic influences, we take it.

In case XXIV. we find that the patient consulted Dr. Flint by letter, in 1859; was relieved of part of her duties as a teacher; took more out of door exercise; traveled in the summer. In 1862 had abnormal dullness, feeble respiration, and increase of resonance and whisper at the right summit. Afterward traveled in Europe; in 1868 had hæmoptysis; in 1869 increase of symptoms, and she died in the sping of 1871. Did this patient recover, and that without change of hygienic condition?

CASE IV.—A physician, aged 28, had hæmoptysis in October, 1852, and again in January and May, 1853. In May, 1853, he had slight dullness at the right summit, with weakened respiratory murmur and crackling, which accompanied inspiration and expiration. In September, 1854, he reported himself well, "a year after recovery:" i. e., his recovery must have dated from September, 1853, five months only after the above symptoms were noted. However, we find that in September, 1854, there was still "dullness at the right summit, and the respiratory murmur was feeble."

CASE XIV.—A physician, who had cough, hæmoptysis and slight loss of flesh, was examined October, 1857, and found to have evidences of phthisis at the left apex. Five years later Dr. Flint saw him in apparently good health, He had been drinking beer, living generously, with an abundance of exercise out of doors. It seems that in this case and in number IV, no medicinal agent was used, though the patients were physicians. They both had the influence of riding and driving in the open air while engaged in country practice.

The three remaining cases do, so far as recorded, seem to be instances of recovery from phthisis without medical or hygienic agency.

Number VIII was a clerk, examined in August, 1856, having had cough two months previously and hæmorrhage a week before. There was dullness and feeble respiration at the right apex and sub-crepitant râles on both sides. In October, 1856, he was reported well, and was in good health in 1871. The thought is at once suggested, why did he, being examined at a time of greatest danger, not have medicinal treatment? The case is, however, one of great interest, and is certainly an exception to the general rule, as the disease appeared, progressed and abated within a period of five months.

The other two cases, XX and XXIII, furnish the best evidence in favor of self-limitation, though the record is very short. Two sisters, whose parents, three sisters and two brothers, had died of phthisis, were found, one with disease of the left apex, the other of the right. No remedy of importance was given, or change made in the habits of life. Both were well 15 years afterward. Again the question may be asked, why was not some form of treatment or change of condition ordered in these cases, as "no effort had been spared to save the lives of their sister and brothers; traveling, changes of climate, together with remedies having been resorted to in vain, although, perhaps, with the effect of retarding the progress of the disease." With this, however, we have nothing to do. The record is that these two sisters for whom nothing was done, alone recovered. Granting that these two cases and the one and possibly the three preceding ones show evidences of self-limitation, yet, we again quote from Prof. Flint's paper, "Self-limitation cannot be inferred from a single case or a very few cases."

Twenty years ago Prof. Flint advanced the idea of the self-limitation of phthisis (*Am. Jour. Med. Sci.*, 1858). A year ago the question was ably reopened by the same author. The conclusions made, though much quoted, have not as yet, as far as we know, been indorsed by any of the many observers and investigators of pulmonary disease. Is it possible that in all this time all others have overlooked this most important element in the progress of phthisis, to which attention has so long ago been called? Does not the etiology, pathology and termination of phthisis plainly teach that without extraneous influence, phthisis is progressive, limited only by death? Clinical evidence is certainly opposed to the doctrine of self-limitation, for we find that even in the large experience of its only advocate, among hundreds of recorded cases, that this doctrine is sustained by but few, and in all fairness we confess a doubt as to the pertinence and value of most of these.

As Prof. Flint has placed the evidence before us, it is the right and duty of every physician to examine it, and to judge for himself. If phthisis is self-limiting, let it be proven so, for with the assertion comes the *onus probandi.* As yet we must believe that self-limitation is not an element in the character of the disease.

Though unwilling to admit that there is an intrinsic tendency in phthisis to recovery, yet we should not lose sight of the fact that many cases of phthisis respond to judicious treatment and hygienic conditions. In truth, in no other disease is careful scrutiny and constant care more necessary or better rewarded. Even were self-limitation ten times proven, it should not lessen the vigilance and attention which such cases demand. In all cases of phthisis two forces are at work—one aggressive and morbid with (as we believe) a direct tendency to death; the other defensive and systemic, opposed to and retarding the progress of disease. Whatever method of treatment is pursued, it and everything connected with it should tend to the building up of the system, and the increase of the powers of resistance to disease. In this we believe is the true limitation of phthisis.

---

MESSRS. DECAISNE, of Paris, have made certain experiments upon the head of a decapitated criminal, which demonstrate that the popular ideas respecting the survival of consciousness after the act of execution, are totally erroneous. The traditions which teach that certain heads have exhibited phenomena, in the winking of eye-lids, biting of lips, blushing, etc., belong evidently to the category of the ghost stories of the nursery. The Messrs. Decaisne, although experimenting upon the head immediately after the descent of the fatal axe, were unable to elicit the faintest evidence of vitality.

